# How to decide which are the most pertinent overly-represented features during gene set enrichment analysis

**DOI:** 10.1186/1471-2105-8-332

**Published:** 2007-09-11

**Authors:** Roland Barriot, David J Sherman, Isabelle Dutour

**Affiliations:** 1 CBiB, Bordeaux Bioinformatics Center, Université Victor Segalen Bordeaux 2, 146 Rue Léo Saignat, 33076 Bordeaux, France; 2 LaBRI, Laboratoire Bordelais de Recherche en Informatique, Université Bordeaux 1, 351 Cours de la Libération, 33405 Talence cedex, France; 3 ESAT-SCD, Katholieke Universiteit Leuven, Kasteelpark Arenberg 10, B-3001 Leuven-Heverlee, Belgium; 4 INRIA Futurs - MAGNOME team - LaBRI, Université Bordeaux 1, 351 Cours de la Libération, 33405 Talence cedex, France

## Abstract

**Background:**

The search for enriched features has become widely used to characterize a set of genes or proteins. A key aspect of this technique is its ability to identify correlations amongst heterogeneous data such as Gene Ontology annotations, gene expression data and genome location of genes. Despite the rapid growth of available data, very little has been proposed in terms of formalization and optimization. Additionally, current methods mainly ignore the structure of the data which causes results redundancy. For example, when searching for enrichment in GO terms, genes can be annotated with multiple GO terms and should be propagated to the more general terms in the Gene Ontology. Consequently, the gene sets often overlap partially or totally, and this causes the reported enriched GO terms to be both numerous and redundant, hence, overwhelming the researcher with non-pertinent information. This situation is not unique, it arises whenever some hierarchical clustering is performed (*e.g*. based on the gene expression profiles), the extreme case being when genes that are neighbors on the chromosomes are considered.

**Results:**

We present a generic framework to efficiently identify the most pertinent over-represented features in a set of genes. We propose a formal representation of gene sets based on the theory of partially ordered sets (posets), and give a formal definition of target set pertinence. Algorithms and compact representations of target sets are provided for the generation and the evaluation of the pertinent target sets. The relevance of our method is illustrated through the search for enriched GO annotations in the proteins involved in a multiprotein complex. The results obtained demonstrate the gain in terms of pertinence (up to 64% redundancy removed), space requirements (up to 73% less storage) and efficiency (up to 98% less comparisons).

**Conclusion:**

The generic framework presented in this article provides a formal approach to adequately represent available data and efficiently search for pertinent over-represented features in a set of genes or proteins. The formalism and the pertinence definition can be directly used by most of the methods and tools currently available for feature enrichment analysis.

## Background

The combination of sequencing and post sequencing approaches together with annotations efforts and *in silico *analysis have produced a tremendous amount of available biological data and knowledge. As technologies evolve, the production of raw data is now becoming daily routine. While transcriptomics produce lists of differentially expressed or co-regulated genes, proteomics produce lists of proteins that are differentially expressed, that carry unusual post-translational modifications or that interact to form a complex. The characterization of those sets of genes or proteins in the light of all available knowledge is therefore a crucial task for the biological researchers and the computational biologists.

To characterize sets of genes or proteins, many tools and methods have been developed (see [1] for a review of most of them) and their main principle is to look for over-represented or enriched features.

Undoubtedly, the key to the success of this technique is its ability to confront heterogeneous data: the set of genes of interest can be compared to the sets of genes i) having the same annotation (*e.g*. Gene Ontology [2] or keywords from UniProt [3]), ii) involved in the same pathway (*e.g*. KEGG Pathways [4]), iii) co-cited in the literature, iv) co-localized on the chromosome, and so on.

**Figure 1:**
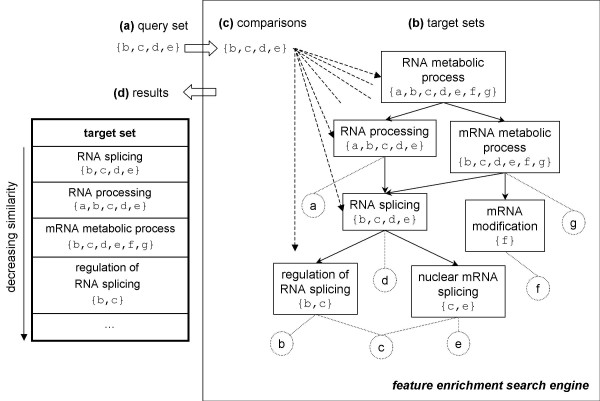
**The processing of a query in a feature enrichment search engine**. (a) a query set is submitted to search for similar sets in (b) a set of target sets. Sets can include each other: this is represented by a graph in which nodes represent sets and edges indicate the inclusion of a set into another. (c) the query set is compared to all the target sets based on a similarity model. (d) target sets found similar are returned ordered by decreasing similarity.

A typical analysis is illustrated in figure 1 through a synthetic example where a query set of genes of interest (the genes b, c, d and e) is searched for enrichment in annotations (an ontology on RNA metabolic process inspired by the Gene Ontology). To process the query, the search engine converts the annotations (genes associated to terms of the ontology) into target sets. For example, the term 'RNA splicing' will be converted to the target set including the gene d which is directly annotated with this term and also the genes that are annotated with the more specialized terms *i.e*. the genes b and c annotated with 'regulation of RNA splicing' and the genes c and e annotated with 'nuclear mRNA splicing' resulting in the target set {b, c, d, e}. During the search, the query set is compared to the target sets by the means of a similarity measure (generally, a dissimilarity index based on a statistical model) and the system returns the similar target sets (hits) with their annotations ordered by decreasing similarity up to a certain threshold. From the enriched features, our query set can be characterized by the RNA splicing process.

Despite the variety of methods proposed to perform such an analysis, very little has been done in terms of formalization, and this has unfortunate consequences. First computationally, the lack of formalism offers very few possibilities for reusable optimizations causing a waste of resources (tool developers time, computation costs and storage space). Considering the growing rate of data, this might soon become an issue. Second and more importantly for the users, current methods generally ignore the structure of the confronted data which leaves the user with numerous enriched features of varying relevance to manually filter and synthesize.

**Figure 2:**
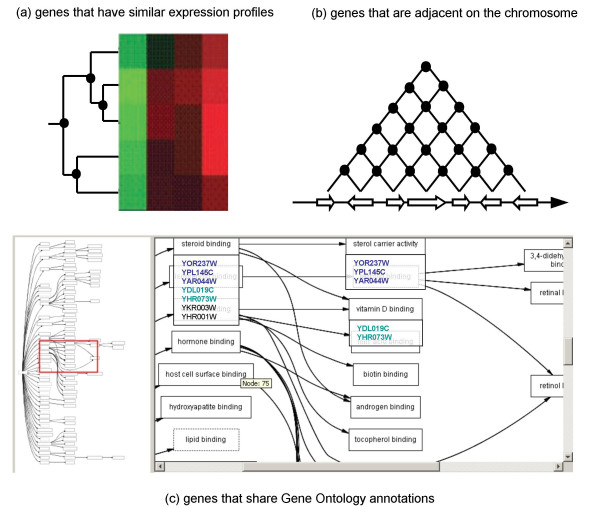
**Examples of neighborhood relationships and target sets**. (a) Expression profiles: target sets correspond to sets of genes having similar expression profiles, *i.e*. nodes of the binary tree resulting from a hierarchical clustering of the profiles. (b) Chromosome localization: sets of adjacent genes correspond to the nodes of an implicit lattice resulting from the order of the genes on the chromosome. (c) Gene Ontology annotations: target sets correspond to GO terms, *i.e*. genes are grouped in a set corresponding to a term when they are annotated with this term or a more specific one.

In this article, we first propose a formalism to represent the feature data. Central to all enrichment search methods is the concept of *neighborhood *proposed by Danchin in [5]: instead of considering genes and proteins as individual entities, the principle is to focus on the relationships between these biological objects (see also [6] for a brief introduction). The generalization of this concept leads to build target sets of entities (*i.e*. sets of genes or proteins) sharing a particular relationship (figure 2). Depending on the relationship, larger target sets can include smaller ones and this information can be represented by directed acyclic graphs [7]. Such directed acyclic graphs (DAGs) are equivalent to mathematical objects: partially ordered sets (posets) which make them perfectly suited for abstraction, formal representation and manipulation. In this paper, we define a neighborhood as the feature data represented by a partially ordered set of target sets.

Based on this formalism, we are able to face the problem of the relevance of the enriched features and the structure of the data, for which we introduce the concept of the *pertinence of target sets *in the context of a given query set of genes. In our synthetic example of figure 1, we observe a redundancy in the hits composing the results: RNA splicing, RNA processing, mRNA metabolic process, etc, are all reported and it is noticeable that RNA splicing matches exactly the content of our query, and therefore only this particular hit should be presented to the user. The other hits are due to the hierarchical structure of the annotations and should be omitted: RNA processing (directly above RNA splicing in the ontology) appears in the results because it includes RNA splicing and another gene (the gene a) not present in the query which should make this target set not pertinent. Similarly, regulation of RNA splicing (directly below RNA splicing) appears because it is included in RNA splicing but contains fewer genes of our query, and this, again, should make this target set not pertinent. These observations allow us to formally define the pertinence of a target set in the context of partially ordered sets. For simplicity here, the target set matches exactly the query, but it is rarely the case, and more than one target set can be pertinent as we explain later. Interestingly, this definition of pertinence holds for the various dissimilarity indices used by current methods (hypergeometric distribution or Fisher's exact test, binomial distribution, *χ*^2^, and percentage). Having a formal pertinence definition, we solve a classical query optimization problem involving a time-space trade off and an early pattern evaluation: instead of storing a very large number of target sets (possibly infeasible), we need to generate only the interesting ones on the fly from a less explicit representation. In this paper, we present algorithms working on compact representations of the data for the generation and evaluation of pertinent target sets. Compact representations exploit the structure of the data (*i.e*. the set inclusions) and algorithms efficiently use rules derived from the pertinence definition.

## Results and discussion

In this section, we first provide formal definitions of neighborhoods (*i.e*. feature data) and target sets pertinence. Then, we introduce an algorithm for the identification and the comparison of pertinent target sets in a DAG when all the set compositions are directly available (like in figure 1b where sets corresponding to DAG nodes are explicitly stored). Next, we propose a generic compact representation of neighborhoods and detail the adaptation of the previous algorithm in this context. The rest of this section focuses on specific representations and algorithms relying on the DAG properties that lead to further time and space optimizations.

### Definitions

#### Uniform representation of data: DAGs defining sets partially ordered by the inclusion relation

We will denote *S *the set of objects considered in the remaining of this paper. For example, *S *can be the set of proteins of an organism. We consider that a neighborhood is a set *N *(of sets of elements of *S*) partially ordered by the inclusion relation ≺. Partially ordered sets (posets) are generally represented by Hasse diagrams in which there is an edge from *y *to *x *if and only if *y *covers *x *(denoted *x *≺ *y*). This means that *x *≺ *y *and there is no other element *z *such that *x *≺ *z *≺ *y *(see [8] for more details).

In the following, we consider that a neighborhood is a Hasse diagram (the DAG of figure 1b) that defines a poset (*N*, ≺).

#### Target sets pertinence

Several methods and tools use a *similarity *or *dissimilarity index *to compare sets, we can cite amongst others: FunSpec [9], BlastSets [7], GOStat [10], EASE [11], PANDORA [12], aBandApart [13], goCluster [14], see [1] for a review. Even though, these methods are using various similarity indices to compare the query and target sets (hypergeometric, binomial, *χ*^2^, Fisher's exact test, or percentages), they all have in common that they consider only counts of elements such as the sizes of the query and target sets, or the number of common and differing elements. Thus, when comparing two sets, the bigger the number of common elements and the smaller the number of differing elements, the more similar they are considered.

Formally, this corresponds to any similarity index *F *between a query set *Q *and a target set *T*, such that *F*(*Q*, *T*) increases with |*T *∩ *Q*| and decreases with |*T *- *Q*|. Then, given such a similarity index for the comparison of a given query set to a neighborhood, it is not necessary to perform the comparisons with all the target sets in the neighborhood. We introduce the notion of *pertinence of a target set *for its comparison to a given query set, which allows to consider target sets that are likely to have good similarity values (elements in common with the query) and to ignore target sets that will give redundant results (not different enough from other target sets because of the set composition dependencies in the Hasse diagram representing the neighborhood).

**Figure 3:**
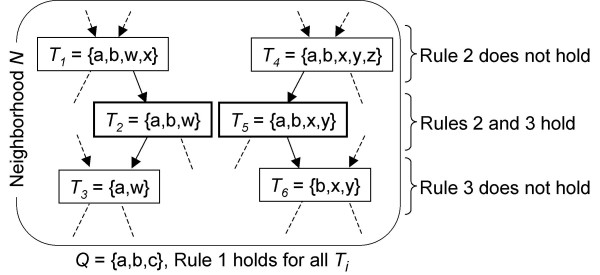
**Illustration of the pertinence rules**. Rule 2 allows the selection of smaller sets containing the same elements in common (fewer differing elements): for *T*_1 _and *T*_4_, Rule 2 does not hold because there exists smaller sets (*T*_2 _and *T*_5_) containing the same elements in common. Rule 3 allows the selection of bigger sets containing the same differing elements: for *T*_3 _and *T*_6_, Rule 3 does not hold because there exists bigger sets (*T*_2 _and *T*_5_) containing the same differing elements. *T*_2 _and *T*_5 _are pertinent.

Our main observation is that redundancy is caused by two target sets when one *includes *the other and when they have either the *same common elements *or the *same differing elements *with the query. For example in figure 3, the target sets *T*_1 _and *T*_3 _are both redundant with *T*_2 _for the query set *Q*. *T*_1 _is redundant because it includes *T*_2 _and has the same common elements (which implies that it has more differing elements) so it is less similar and it does not bring more information than *T*_2 _alone. Similarly, *T*_3 _is redundant because it is included in *T*_2 _and has the same differing elements (*i.e*. less common elements). The fact that one target set includes the other is important for the biological meaning of the results. Let us consider *T*_2 _and *T*_5 _of figure 3. In this case, one should be tempted to decide that only *T*_2 _is pertinent (same common elements and fewer differing elements). Actually, *T*_5 _is also pertinent because the differing elements do not include those of *T*_2 _and thus *T*_5 _may be associated to a pertinent nonredundant biological meaning.

Mathematically, the observation above is written simply as follows:

**Definition**. *A target set T in a neighborhood N is *pertinent *for its comparison to a given query set Q if and only if:*

*T *∩ *Q *≠ ∅

∄*T' *∈ *N *such that *T' *⊂ *T *and *T' *∩ *Q *= *T *∩ *Q*

∄*T' *∈ *N *such that *T' *⊂ *T *and *T' *- *Q *= *T *- *Q*

This mathematical definition suggests that one must test all possible *T' *to decide if a target set *T *is pertinent. However, it is easy to deduce that only the parent and child nodes of *T *in the Hasse diagram representing the neighborhood *N *must be checked. Let us suppose that such a *T' *exists for (2) (resp. (3)). Then, due to the inclusion relation between *T *and *T'*, all the sets in *N *on the path linking *T *and *T' *also satisfy (2) (resp. (3)), and then especially a child node (resp. a parent node) of *T*.

As only the parent and child nodes of *T *need to be considered, the test of pertinence can be performed on the *number *of common and differing elements. This is because if these numbers are equal then we are in presence of the same elements (inclusion relation).

As a result, the mathematical definition can be simplified into the following 3 rules (illustrated in figure 3) that are more suitable for the design of an efficient algorithm:

**Rule 1: **|*T *∩ *Q*| ≠ 0

**Rule 2: **∄*T' *such that *T' *≺ *T *and |*T *∩ *Q*| = |*T' *∩ *Q*|

**Rule 3: **∄*T' *such that *T *≺ *T' *and |*T *- *Q*| = |*T' *- *Q*|

### Structures and algorithms

#### Algorithm for the identification and the comparison of pertinent target sets in the explicit representation

**Figure 4:**
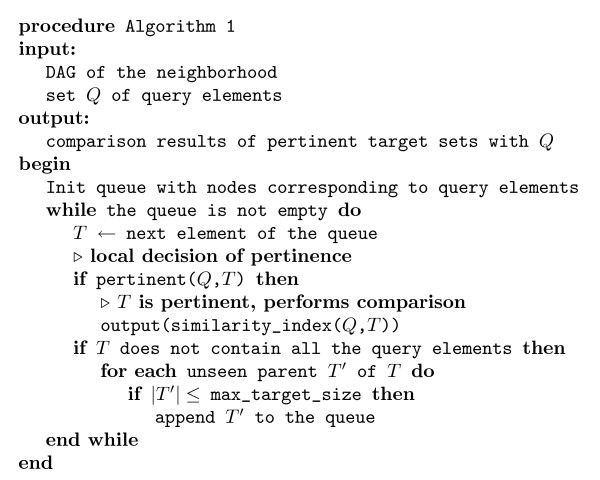
**Algorithm 1**. Algorithm 1 for the identification of pertinent target sets in the DAG of a neighborhood (see figure 1b) and their comparison to a given query set. It is a slightly modified version of a multiple sources breadth first search.

**Figure 5:**
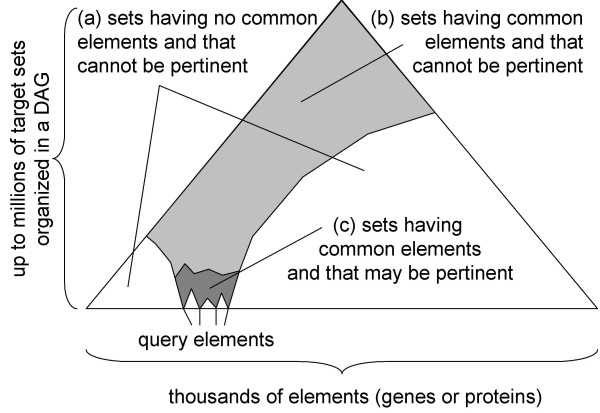
**Pertinence depends on query elements**. Only a small fraction of all the target sets may be pertinent for a given query: (a) sets that have no elements in common with the query are not pertinent. (b) large sets (much bigger than the query) having elements in common with the query are not pertinent because they contain too much differing elements. (c) small target sets that have elements in common with the query may be pertinent.

The pertinence rules allow us to define an algorithm (given in Algorithm 1 of figure 4) for the identification of target sets that are pertinent for their comparison to a given query set. Its principle is to search the DAG of the neighborhood, starting from the leaves corresponding to query elements (Rule 1) and exploring their ancestors to identify nodes satisfying the pertinence definition. This corresponds to a multiple sources breadth-first search, in which the queue is initialized with the nodes corresponding to the query elements. Each time a node is processed it is tested for pertinence. The search can stop at nodes including the query (Rule 2) or when the target set size is too big to give a significant similarity value. In the latter case, a test on the target set size is performed if an upper bound max_target_size can be computed theoretically (which is the case for most of the similarity models). In this algorithm, we suppose that the sets corresponding to the nodes of the DAG are available (which is not always feasible). The worst-case time complexity of a breadth-first search is *O*(*V *+ *E*) where *V *is the number of vertices of the DAG and *E *is the number of edges. To test the pertinence of a node, we need (i) to compute the number of common and differing elements of the sets corresponding to the nodes, and (ii) to compare these values to the parent and child nodes to test if neither Rule 2 nor Rule 3 are violated. The computation of the number of common and differing elements for a node can be done in *O*(|*S*|), the maximum size of a set. The test of pertinence done in pertinent(*Q*, *T*) necessitates an access to all the parent and child nodes, which adds up to *O*(2*E*) supplementary tests. Thus, the worst-case time complexity of Algorithm 1 is *O*(|*S*|*V *+ 3*E*) = *O*(|*S*|*V *+ *E*). The worst-case time complexity occurs when all the nodes except the root include some but not all of the query elements. Let us consider the average case, in which we expect the query set to be small compared to the total number of elements |*S*|. The target sets sharing elements with the query represent only a subgraph of the DAG (figure 5b and 5c), and, the pertinent target sets should have sizes that are commensurate with the query size, which implies that they are deep in the DAG (figure 5c). Then, the number of nodes processed is typically very small compared to *V*. Moreover, the average target set size is small compared to |*S*|. Thus, the added |*S*| factor to the complexity may be considered as a constant and be negligible in the average case.

Algorithm 1 assumes that the set compositions are available. In the following, we introduce compact representations of neighborhoods and algorithms efficiently working on such representations.

#### Generic compact representation of neighborhoods

**Figure 6:**
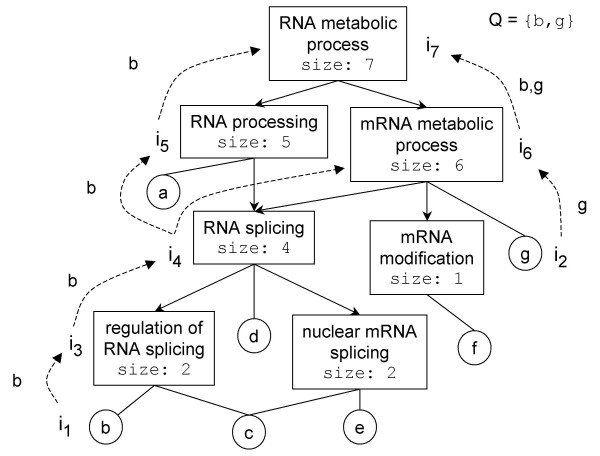
**Generic compact representation of a neighborhood**. Instead of explicitly storing all the set compositions as illustrated in figure 1b, only the size of the set corresponding to a node is stored. Nodes corresponding to singletons sets (leaf nodes) are labeled with the element of the set. The set corresponding to a particular node can be generated on the fly by searching the leaf nodes reachable from this set. During the bottom-up search of pertinent target sets, elements in common with the query set *Q *= {*b, g*} are propagated. i_1_..i_7 _correspond to iterations of the main loop (order in which the nodes are processed).

It is generally inefficient to explicitly store the composition of all the sets of a neighborhood. A compact representation is needed. Such a representation should permit one both to identify and to generate pertinent target sets efficiently, in a way that avoids the generation of all the sets and the traversal of the entire graph. Indeed, the uniform representation of neighborhoods by DAGs is adequate for compactness: we can store only the DAG defining the poset and reconstruct sets corresponding to nodes on the fly. In this compact representation, leaf nodes (nodes without successors) correspond to singleton sets (one for each element of *S*) and all the other nodes correspond to sets that can be built by searching the labels of reachable leaf nodes. For efficiency reasons, internal nodes are labeled with the size of the sets they represent as we will explain later. Figure 6 illustrates the compact representation corresponding to the DAG of figure 1b.

#### Algorithm for the identification of pertinent target sets in the generic compact representation

Like in Algorithm 1, the principle is to start from the leaves representing query elements and traverse the DAG to search for pertinent target sets among the sets sharing elements with the query. The difficulty we have to solve is that the set compositions are not available. We thus (i) store the size of the set corresponding to a node, as illustrated in figure 6, (ii) order the nodes in the queue by their size and (iii) propagate the common elements during the search.

In order to test Rule 2, we need the number of common elements of the node processed and its child nodes. With the breadth-first order, the node 'mRNA metabolic process' of size 6 of figure 6 would have been processed before the node of size 4 'RNA splicing' (shorter path from the leaves), and thus the common element *b *would not have been propagated yet. The solution is to maintain the queue ordered by the set sizes to ensure that sets smaller than the node processed (this includes all its descendants) have already been processed. This way, all the common elements have been propagated and Rule 2 can be tested at the level of the node processed.

In order to test Rule 3, we need the number of differing elements of the node processed and its parent nodes. Unfortunately, this number is not available at this time for the parent nodes because all the common elements may not have been propagated to the parent nodes yet. For example, the node 'RNA processing' (size 5) in figure 6 is processed before the element *g *has been propagated to the node 'RNA metabolic process' of size 7. The solution is to consider the node processed as the parent node and test if Rule 3 is not violated for its child nodes that is, we test the pertinence of its child nodes.

As a result, the pertinence decision is divided in two steps:

**Step 1: **Rule 2 is tested at the level of the node processed.

**Step 2: **Rule 3 is tested at the level of the child nodes of the node processed.

**Figure 7:**
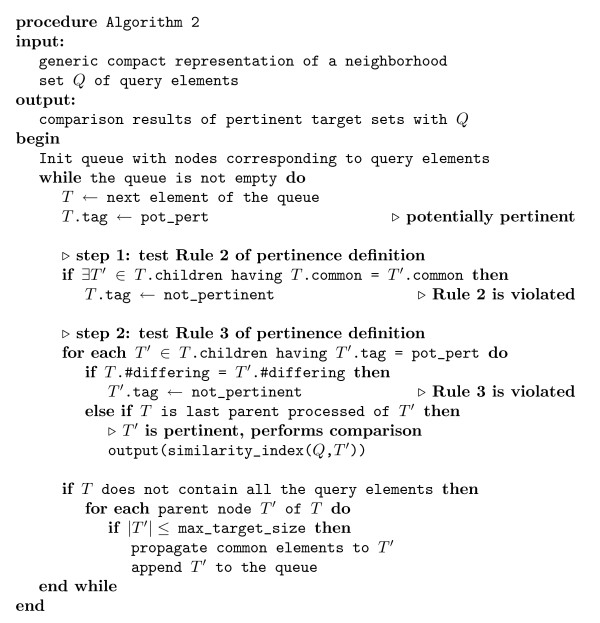
**Algorithm 2**. Algorithm 2 for the identification and the comparison of pertinent target sets in the generic compact representation (see example in figure 6) for their comparison to a given query set. The node data structure has the following fields: tag for the potentially pertinent tag, children and parent that point to child and parent nodes, common which is the set of common elements with the query, #differing for the number of elements differing with the query set, size for the size of the set this node represents.

The efficiency of the resulting algorithm (given in Algorithm 2 in figure 7), compared to Algorithm 1, is only affected by the extraction of the next element of the queue, which must be ordered by the set sizes. As we can have at most |*S*| different sizes of set, the worst-case time complexity is affected by a factor of log |*S*| by using an adequate data structure. As for the previous algorithm, the average target set size is expected to be small compared to |*S*|, so the log |*S*| factor may be considered as a constant and be negligible.

#### Specific representations of neighborhoods

The previous compact representation is general and can be used for any neighborhood. Nonetheless, we identified cases where further time and space optimizations can be envisaged. It arises when:

• the DAG is actually a *tree *(each node has only one ancestor). In this case, the tree can be stored as a parenthesized expression without the need to store the size of the sets for each node. Typical examples of this situation correspond to the gene expression profiles hierarchically clustered, or the IUBMB Enzyme Nomenclature [15] that is, sets of genes/proteins annotated with the same EC number.

• the DAG obtained after building the neighborhood is *implicit *and thus, does not need to be stored. It corresponds for example to a correspondence analysis or a principal component analysis of the codon usage (see [16]) or the sets of genes that are adjacent on the chromosome. In the latter case, we only need to know the order of the genes: any pair of genes defines an interval which defines a set of adjacent genes.

In the following, we present efficient algorithms for the identification of pertinent target sets in these specific representations.

#### Algorithm for the identification and the comparison of pertinent target sets in the tree compact representation

The main advantage to searching pertinent target sets in a tree is that for a given node, the child nodes define a non overlapping partition of the set their parent node represents, and thus, only the number of common and differing elements need to be propagated.

The principle is to recursively compute a triplet of values (number of common elements, number of differing elements, tag indicating potential pertinence) for each node by using a stack of stacks to parse the parenthesized expression. The idea is to push an empty stack when an opening parenthesis is encountered, or a triplet of values when an element is encountered. When a closing parenthesis is read, the computation can occur and consists in the following:

(i) Compute the number of common and differing elements corresponding to this node by summing up the values of the triplets contained in the top stack.

(ii) Test Rule 2 for current node: if the number of common elements is bigger than all of the triplets contained in the top stack then the tag is set to potentially pertinent (not pertinent otherwise).

(iii) Test Rule 3 for child nodes: if child nodes tagged potentially pertinent have less differing elements than the current node, then they are pertinent and the comparison is performed.

(iv) Replace the top stack by the triplet of computed values.

(v) Stop if all the query elements are included or if the target set size exceeds max_target_size.

Compared to the previous algorithm, this one avoids (i) the merging of the common elements for each node and (ii) the extraction of the next element of the queue. The tree is composed of |*S*| leaves and at most |*S*| - 1 nodes, thus, the worst-case time complexity of this algorithm is *O*(|*S*|).

#### Algorithm for the identification and the comparison of pertinent target sets in implicit compact representations

An implicit representation requires us to provide a specific algorithm for each different implied DAG. However, this loss in genericity allows considerable time saving in the search for pertinent target sets and considerable space savings for storing the neighborhoods. We have chosen to present the sets of adjacent genes on the chromosome because it can be described very simply and briefly, leads to a straightforward algorithm, and was often encountered in our experiments.

For our illustration, we only need to store the genes in the order they appear on the chromosome. Thus, the space requirement is *θ*(|*S*|), instead of *θ*(|*S*|^2^) needed for the DAG representation.

To identify pertinent target sets, we only need to know the position on the chromosome of each of the query elements. Then, each pair of positions defines a lower and an upper bound of an interval that, in turns, defines a set. For such a set to be pertinent, the bounds of the interval must be such that the position just before (resp. after) the lower (resp. upper) bound must not be an element of the query since it would violates Rule 3. Rule 2 holds because the bounds correspond to query elements. The worst-case time complexity of the resulting algorithm is *O*(|*Q*|^2^), *Q *being the query set. Compared to Algorithm 2 working on the generic compact representation, this algorithm spares (i) the merging of common elements and (ii) the extraction of the next element of the queue.

## Testing and validation

In this section, we illustrate the gain in storage space, number of comparisons performed and quality of the results (redundancy reduction) through a typical search of Gene Ontology [2] annotations enrichment in sets of proteins corresponding to multi-protein complexes, and compare the results obtained with and without considering the pertinence of target sets. For convenience, we used the BlastSets system [7] to obtain these results because it allows to use all the sets of a neighborhood (protein complexes) as query sets to be searched for feature enrichment (Gene Ontology annotations), but any of the previously cited methods and tools may be used instead.

### Data sets

#### Query sets

The query sets of proteins correspond to protein complexes of the yeast *Saccharomyces cerevisiae *referenced at the MIPS [17] (version 14112005 filtered against a list of validated open reading frames from the GDR Genolevures [18]). The motivation for this choice is that once the proteins involved in a complex are identified, the next step is often to search for a molecular function or a biological process with which to annotate the newly grouped set of proteins. Moreover, the yeast proteome is well annotated with 4 211, 4 936 and 5 451 annotated gene products (among about 6 000) respectively for the molecular function, biological process and cellular component branch of the Gene Ontology (source: [19]). We extracted 1062 query sets of proteins, one for each protein complex.

#### Target sets/neighborhoods

The Gene Ontology is organized in a DAG hierarchical structure. The pertinence definition is thus perfectly suited to this case as GO terms allow very specific as well as very general annotations, which may often lead to results of varying relevance. We constructed three Gene Ontology neighborhoods (GO version 2005-03-01, and gene associations using all evidence codes except IEA), one for each Gene Ontology branch *i.e*., cellular components (denoted CC), molecular functions (MF) and biological processes (BP), by performing the following:

(i) the DAG of the Gene Ontology is the generic compact representation of the neighborhood,

(ii) to the previous DAG, we add (leaf) nodes corresponding to proteins, and connect them as child nodes for each GO term they are annotated with,

(iii) we recursively traverse the DAG in a bottom-up fashion to compute the size of the set corresponding to each node.

This construction implies that when a protein is annotated with a GO term, all GO terms on the paths from this term to the root (more general terms) are also annotating this protein.

### Overall validation performances

Each of the 1 062 protein complexes served as a query set of proteins, and each was searched for similar sets in the three Gene Ontology branches constructed neighborhoods. The threshold for set similarity significance was set to 0.05. This corresponds to the probability of obtaining a similarity value (here *F *is the hypergeometric distribution) at least as good by submitting a random set of the same size, see [7] for more details.

#### Space requirements

We compared the storage space needed by the generic compact representation (figure 6) and by the explicit storage of sets compositions (figure 1b). We assume that the set sizes, nodes and elements identifiers are represented by the same memory unit (MU), say 32 bit integers. For the explicit representation, the sets are represented as lists associated with nodes identifiers. For the generic representation, the size of the sets are stored directly in adjacency lists. The sizes obtained are listed in table 1. As a result, the space needed to store the generic representation requires only 27% to 49% of the space needed for the explicit representation. Note that the DAGs have small sizes (308, 756, 949, 2 103 nodes for respectively CC, MF, BP and combined neighborhoods). Thus, in this situation, an explicit representation is not prohibitive. However, it is noticeable that even for such small sizes, the compact representation requires at most half of the storage space needed by the explicit representation. As the sizes of these DAGs will grow (larger *S *and/or new GO annotations) the generic representation performances will increase accordingly.

**Table 1:** Storage space requirements results

	Cellular Components	Molecular Functions	Biological Processes	Combined
explicit size in MU (figure 1b)	48, 862	32, 187	72, 947	154, 839
generic size in MU (figure 6)	16, 611	15, 734	20, 317	41, 136

ratio generic/explicit	34%	49%	28%	27%

Summary of the results obtained for the 3 Gene Ontology branches extracted neighborhoods (CC, Cellular Components; MF, Molecular Functions; BP, Biological Processes) in terms of space requirements in memory units (MU, *e.g*. 1 MU = 32 bits) for the explicit (sets compositions are stored) versus the generic representation of neighborhoods.

#### Efficiency

Table 2 gives a summary of the number of comparisons required to search for similar sets: (i) all the query sets are compared to all the target sets, and (ii) only the pertinent target sets are compared. Among all the possible comparisons, only 1.11% to 2.07% are relevant. A simple improvement would be to compare only target sets having elements in common with the query. Even in this case, we see that the pertinence definition allows to perform only 23% to 39% of the comparisons that would have been done with sets having common elements with the query.

**Table 2:** Efficiency results

	Cellular Components	Molecular Functions	Biological Processes	Combined
all comparisons	327, 096	802, 872	1, 007, 838	2, 137, 806
common elements comp.	19, 821	22, 556	48, 084	90, 461
pertinent target sets comp.	6, 769	8, 884	11, 440	27, 093

ratio pertinent/all	2.07%	1.11%	1.13%	1.26%
ratio pertinent/common	34%	39%	23%	29%

Summary of the results obtained for the 3 Gene Ontology branches extracted neighborhoods in terms of efficiency that is, the number of sets comparisons performed.

#### Redundancy reduction

In table 3, we give, for each GO branch, the number of sets found significantly similar (hits) to protein complexes with and without applying the test of target set pertinence. These results show that pertinent target sets similar to the query represent only 50%, 48% and 36% of the hits returned by the system. Thus, with at least half less results to examine, the analysis and interpretation made by biologists is simplified and more effective.

**Table 3:** Redundancy reduction results

	Cellular Components	Molecular Functions	Biological Processes	Combined
hits	1, 842	1, 473	3, 381	6, 696
pertinent hits	922	703	1, 205	2, 830

ratio pertinent hits/hits	50%	48%	36%	42%

Summary of the results obtained for the 3 Gene Ontology branches extracted neighborhoods in terms of redundancy reduction that is, the number of target sets found similar (hits) to the 1062 MIPS protein complexes in the Gene Ontology obtained with and without applying the test of pertinence.

### Typical outcome for a protein complex

**Figure 8:**
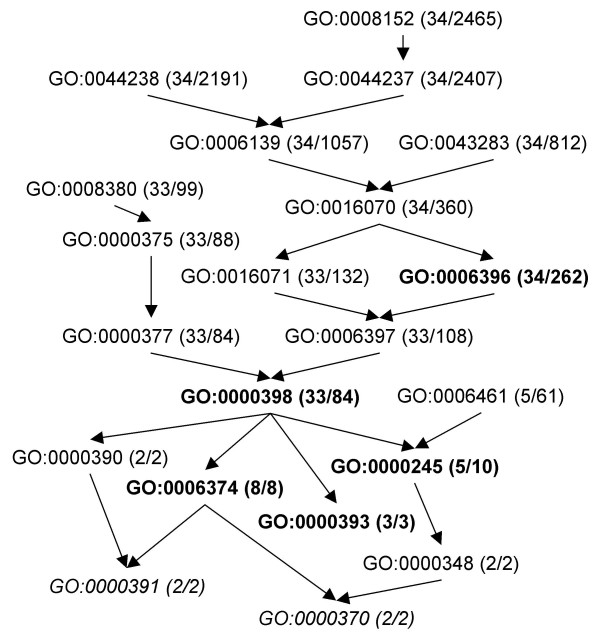
**Part of the Gene Ontology DAG containing hits listed in table 4**. Partial view of the DAG of the Gene Ontology biological processes branch concerning hits of complex 440.30.10 of the MIPS listed in table 4. Pertinent terms are in bold, non pertinent terms due to Rule 2 are in normal shape and non pertinent terms due to Rule 3 are in italic. The number of proteins annotated with the term in the complex is given between parenthesis together with the total number of proteins annotated with this term (target set size).

To illustrate further the gain in results pertinence, we focus on one particular MIPS complex: '440.30.10 mRNA splicing' composed of 36 proteins. The results obtained for this complex emphasize well the redundancy issue and the need of pertinence testing. The list of similar sets found (hits) in the BP branch is given in table 4. The relationships between GO terms are given in figure 8.

**Table 4:** Sets found similar to the MIPS '440.30.10 mRNA splicing' protein complex

GO Term	description	size	common
**GO:0000398**	**nuclear mRNA splicing, via spliceosome**	**84**	**33**
GO:0000377	RNA splicing, via transesterification reactions with bulged adenosine as nucleophile	84	33
GO:0000375	RNA splicing, via transesterification reactions	88	33
GO:0008380	RNA splicing	99	33
GO:0006397	mRNA processing	108	33
GO:0016071	mRNA metabolism	132	33
**GO:0006396**	**RNA processing**	**262**	**34**
GO:0016070	RNA metabolism	360	34
GO:0043283	biopolymer metabolism	812	34
GO:0006139	nucleobase,...	1057	34
GO:0044238	primary metabolism	2191	34
GO:0044237	cellular metabolism	2407	34
GO:0008152	metabolism	2465	34
**GO:0000245**	**spliceosome assembly**	**10**	**5**
GO:0006461	protein complex assembly	61	5
**GO:0006374**	**nuclear mRNA splicing via U2-type spliceosome**	**8**	**8**
*GO:0000391*	*U2-type spliceosome dissembly*	*2*	*2*
GO:0000390	spliceosome dissembly	2	2
*GO:0000370*	*U2-type nuclear mRNA branch site recognition*	*2*	*2*
GO:0000348	nuclear mRNA branch site recognition	2	2
**GO:0000393**	**spliceosomal conformational changes to generate catalytic conformation**	**3**	**3**

Sets found similar to the MIPS '440.30.10 mRNA splicing' protein complex (36 proteins) in the neighborhood constructed on the biological processes branch of the Gene Ontology. Results are sorted to better illustrate pertinence definition application (*i.e*. not by similarity value). Pertinent target sets are in bold shape. Non pertinent target sets that violate Rule 2 are in normal shape (test of Rule 3 not performed). Non pertinent target sets that violate only Rule 3 are in italic.

Among the 21 hits, only 5 are actually pertinent, 14 hits violate at least Rule 2, the test of Rule 3 not being performed, and 2 hits violate Rule 3. The target sets are sorted in order to better understand the violations of pertinence: pertinent target sets are listed first in bold, followed by target sets that are not pertinent because of the previous pertinent set. The first hit, GO:0000398, is pertinent and the following 5 hits are not as they have the same number of common elements but correspond to less specific GO terms, which violates Rule 2. The same scenario occurs for the next 2 pertinent target sets, GO:0006396 and GO:0000245 with respectively 6 and 1 following hits that violate Rule 2. Then, GO0006374 is pertinent, and the following, GO:0000391, is not because it has the same differing elements (none) and has less common elements *i.e*. it is less general, which violates Rule 3.

Only 34 of the 36 query elements are found together in a hit. In the general case, this may (i) highlight bad annotations or (ii) provide a hint or indications on the role of the missing elements. Here, the missing elements are the products of the genes YGL128c and YKL078w. YGL128c is annotated as 'Component of a complex containing Cef1p, putatively involved in pre-mRNA splicing'. It is currently annotated with 'biological process unknown' which explains why it is not found in the results. Interestingly, it is associated with 'spliceosome complex' in the cellular components branch which complies with its supposed involvement in pre-mRNA splicing. Moreover, its association to a complex containing Cef1p strongly suggests that YGL128c should be annotated with GO:0000398. YKL078w is annotated as 'Predominantly nucleolar DEAH-box RNA helicase, required for 18S rRNA synthesis'. It is annotated as GO:0007046 ribosome biogenesis of the biological process branch of the GO. This term corresponds to a set of size 150 that is not part of the results that is, it is not significantly similar to 440.30.10. In [20], the authors state that YKL078w is not required in pre-mRNA splicing, but it is required for pre-rRNA cleavage (18S rRNA synthesis), and thus its GO annotation is consistent.

### Comparison to other methods

Since 2001, several methods (more than 15) have been successfully applied to search for over-represented features based on a dissimilarity index to compare a query set to target sets (most of those are reviewed in [1]). More recently, alternative or complementary approaches have been developed, mainly to find more relevant features or to combine multiple features. Hereafter, we discuss and compare our methods to the major trends in the field.

#### Frequent itemset mining

The problem of identifying pertinent target sets resembles the frequent or closed itemset mining problem (see [21] for details) in many aspects. Unfortunately, the methods developed for frequent itemset mining cannot be applied to our context. Indeed, these methods rely on the anti-monotonic property of the support function (minimum frequency of the itemsets in the database). In our situation, the pertinence test is not anti-monotonic: a non pertinent target set can have ancestors that are pertinent. As a result, the pertinence definition cannot be used in the same way to prune the search. Moreover, closed itemsets permit the generation of all frequent itemsets contrary to pertinent target sets (all their subsets are not necessarily pertinent and may also not be present in the neighborhood).

#### Dissimilarity index based over-representation methods and tools

The methods we describe in this article improve the global quality of the results found by using a statistical test to decide the over-representation of a given feature in a given set (reviewed in [1]). Depending on the test performed (hypergeometric, binomial, *χ*^2^,...) and the correction for multiple testing (bonferroni, false discovery rate,...), the set of over-represented features will vary in size but the top features (very significant *p-values*) will remain essentially the same. Among those features, we clearly showed mathematically and also with biological results that a lot of them are actually redundant and non-informative. As it is based on the same principles (dissimilarity index and adjustment for multiple testing), our method can only perform at least as good as those others. For example, we submitted the query set of proteins of the complex 440.30.10 to GOStats [10] and we obtained 24 significantly enriched GO terms in the biological processes branch among which 7 are not in table 4. The observed differences (additional hits) are due to different versions of the data and to the multiple testing adjustment method (more low similarity hits). The additional hits are related to the pertinent hits found in table 4 and do not bring much additional insight to our query set biological function.

An original approach exploiting the GO structure was proposed in [22]. Like us, they consider the GO as a partially ordered set and work on the DAG. Their method and ours diverge due to the dissimilarity index. To score target sets, they define a pseudo distance which can be stated roughly as the average distance between the genes of the query and the target GO term. While this approach is formal and applicable to ontologies in general, it suffers some significant limitations. First computationally, for each query set they need to score all the GO terms. Second statistically, because of the use of a distance, they are only able to rank the GO terms and cannot assess the significance of the results. And finally, they also encounter the problem of redundancy and pertinence of the results. They partially address it by finding in the top ranked GO terms, the ones that are not comparable (*i.e*. the one that should bring more non redundant information).

#### Information content based methods

An interesting approach has been proposed by [23] to take into account the GO hierarchy. The difficulty to address when dealing with the GO hierarchy is that the level of a GO term in the hierarchy does not reflect the degree of specificity of this term. As a result, the degree of specificity (GO level) at which to look for enrichment should not be specified in the query because it can yield to misleading results or missed discoveries. In [23], the authors propose an information theoretic approach that allows to specify the degree of specificity desired for the enriched features. This is done by splitting the graph of the Gene Ontology into subgraphs. The split is such that the resulting subgraphs (partition of the GO terms) contain comparable information content, *i.e*. they concern the same number of genes. To illustrate their approach, they analyze the 'MAP00190 oxidative phosphorylation' set of proteins corresponding to GenMAPP proteins involved in oxidative phosphorylation. For this analysis, the Gene Ontology was split into 6 partitions among which a clear enrichment in 'transport' is visualized. In contrast, a corresponding GO biological process levelwise analysis performed at depth 2 exhibits visual enrichments in 'cellular process' and 'physiological process' which is misleading.

Results obtained with our method on the same query set, MAP00190 oxidative phosphorylation, are presented in table 5 for enrichments (sorted by *p-values*) in the GO biological process branch. In this table, proton, cation and electron transport appear at the 1^*st*^, 2^*nd *^and 3^*rd *^position respectively. It is worth noting that there exists other significant enrichments not reported in [23] (*e.g*. GO:0006091 generation of precursor metabolites and energy and GO:0006119 oxidative phosphorylation). This is due to the pre-specified level of specificity.

**Table 5:** Pertinent sets found similar to the 'MAP00190 oxidative phosphorylation' consisting of GenMAPP proteins involved in oxidative phosphorylation

GO Term	description	size	common
GO:0015992	proton transport	26	9
GO:0006812	cation transport	180	11
GO:0009060	aerobic respiration	15	5
GO:0006118	electron transport	90	7
GO:0006091	generation of precursor metabolites and energy	209	9
GO:0006119	oxidative phosphorylation	39	5
GO:0006825	copper ion transport	7	2
GO:0006878	copper ion homeostasis	7	2
GO:0006885	regulation of pH	10	2
GO:0050801	ion homeostasis	122	4
GO:0006120	mitochondrial electron transport, NADH to ubiquinone	26	2

With our method, it is not possible to specify a given degree of specificity as with their tool GOPaD [23]. However, similar results can be obtained by constructing other neighborhoods for the Gene Ontology that would correspond to different level of specificity or information content. An alternative solution can also be to use GO Slim (reduced version of the GO aimed at giving an overview of its content) instead of the whole Gene Ontology. More generally, by looking at all the levels of the GO hierarchy, our method successfully identifies pertinent target sets, which automatically selects the most relevant levels to look at. Besides, our method is more generic in the sense that it can be applied to any hierarchically defined sets. For example, it can be applied to the hierarchical clustering of gene expression profiles which results in a dendogram (figure 2a) or the gene localization on chromosomes (figure 2b). In those cases, an information content method is of no help because the degree of specificity needs to be specified *a priori *and this is typically not known.

#### Integration of multiple data sources

Another trend in the field is the search for enrichment in combination of features by the use of multiple data sources. The direct approach consists in intersecting target sets as proposed in [24] where target sets of genes with composite GO annotations are obtained. This allows to find enrichments that are significant for the composite annotation (*e.g*. 'cation transport' and 'ATPase activity') while not being enriched in the original annotations (*i.e*. 'cation transport' alone or 'ATPase activity' alone). A similar approach has been proposed in [25] where frequent co-annotations (keywords, GO terms, and KEGG pathways) are mined. The principle is to search for frequent itemsets in the features of the query genes (features co-occurring frequently), and then to look at the significance of the enrichment in the combined features. Alternatively, the converse approach consists in the addition of GO term relationships such as *is-involved-in *as proposed in [26]. The principle is to augment the Gene Ontology to connect terms from different branches (sub-ontologies) to reflect the fact that a molecular function is involved in a biological process which takes place in a cellular component.

Although, the enrichment of combination of features is not addressed in this paper, similar results can be obtained by manipulating the neighborhoods. For example, it is possible to combine the GO biological process and the GO molecular function neighborhoods by adding nodes corresponding to the set intersections (composite annotations) to the Hasse diagram representing the neighborhood. Similarly, the augmentation of neighborhoods such as the additional Gene Ontology layer proposed in [26] can be achieved by adding the corresponding edges between GO terms and by propagating the gene products through the newly created paths. This could prove useful as we have seen in the results obtained for complex 440.30.10 with the gene YGL128c that the GO annotations are sometimes missing in a particular branch whereas present in another one.

#### Numerical features

Numerical features can be very interesting to consider for feature enrichment. For example, it can be used to discover that some genes of a query set are surprisingly close to each other on a chromosome, or that all the molecular weights of the query proteins fall within a surprisingly small range. To our knowledge, our approach is the sole capable of searching for enrichments in numerical features such as the gene localization on chromosomes (see figure 2a and the results section on implicit compact representations). This might be because (i) it is inefficient and sometimes unfeasible to store and compare all the sets corresponding to adjacent genes and (ii) because the redundancy in the results (if not filtered for pertinence) makes them unexploitable.

## Conclusion

In this article, we addressed the problem of the characterization of a set of genes or proteins by finding pertinent over-represented features. The key advances presented here are a formalism for representing and manipulating the data to be searched, and the introduction of the concept of target set pertinence and its formal definition. The choice of partially ordered sets as a formal representation was naturally driven by the generalization of the concept of neighborhood between genes or proteins: biological relationships (*e.g*. similar expression profile, similar function, similar annotation) group genes or proteins into sets of neighbors, which can be nested. These foundations exhibit their strength in many aspects. First, they make it possible to take into account the structure of the data and get rid of the non informative results. Second, their generic and universal aspect make them directly usable by most of the current methods and tools (the pertinence definition holds for most of the dissimilarity indices in use). Third, they provide a solid basis on which to develop optimized structures and algorithms such as those presented in this article: a generic compact representation applicable to any neighborhood, a specific compact representation for trees (*e.g*. hierarchical clustering of gene expression profiles), and an example of an implicit compact representation for gene location on chromosomes. The validation was performed by searching enriched GO annotations in 1062 protein complexes. The performances observed clearly show the usefulness of our approach: in terms of resources, we were able to save up to 73% storage for the data and to avoid up to 98% of the comparisons performed between sets during the search. More importantly, we observed up to 64% of statistically significant enriched features that were actually not pertinent and that should be discarded. This means that the biological researchers and the computational biologists will be presented far less results to interpret, making the characterization of gene sets faster, safer and easier.

In this article, we illustrated our methods with sets of genes and proteins examples but they can be applied to other data as well: formally, our approach is already general because it only considers elements of a finite set. Good candidate data sets should exhibit a finite set *S *of elements and various neighborhood relationships. These relationships can be inferred for example from a *many to many *relation between elements of *S *and elements of another set, or, a hierarchical structure and a relation associating elements of *S *to nodes of the hierarchy. For example with the growing number of complete genomes available, it should be interesting to build sets of genomes based on various neighborhood relationships and test what features result in similar groupings.

The methods presented in this paper naturally lead to a new challenge: the identification of similar sets between two neighborhoods. Such a task is of utmost importance as it would allow to analyze nearly automatically large amounts of data. For example, for gene expression data (as in figure 2a), the clusters of co-expressed genes would be matched to pertinent Gene Ontology terms. A naive solution would generate all the sets of one neighborhood and submit them as independent query sets to identify similar sets in the other neighborhood. This approach has two significant drawbacks. First, it implies the generation of all the sets of a neighborhood, which is exactly what we sought to avoid, and more importantly, the query set inclusions will cause redundancy both in the computations and in the results. Second, the pertinence definition is not symmetric, that is, given two neighborhoods *N*_1 _and *N*_2_, the results obtained will differ depending on which neighborhood (*N*_1 _or *N*_2_) will serve as the query and the target neighborhood. This is because all the query sets are assumed pertinent, which is typically not the case. In the example of the hierarchical clustering of gene expression profiles and the Gene Ontology, a "two-sided" pertinence definition would allow to identify the *pertinent *clusters to be compared to the *pertinent *GO terms. Thus, the pertinence definition should be reviewed in this context to ideally permit the design of algorithms that search both neighborhoods simultaneously.

## Authors' contributions

RB wrote the computer code, carried out the experiments, and drafted the manuscript. ID drafted and refined the manuscript. DJS and ID both provided substantial comments. All authors read and approved the final manuscript.
